# A Genome-Wide Study of Modern-Day Tuscans: Revisiting Herodotus's Theory on the Origin of the Etruscans

**DOI:** 10.1371/journal.pone.0105920

**Published:** 2014-09-17

**Authors:** Jacobo Pardo-Seco, Alberto Gómez-Carballa, Jorge Amigo, Federico Martinón-Torres, Antonio Salas

**Affiliations:** 1 Unidade de Xenética, Departamento de Anatomía Patolóxica e Ciencias Forenses, and Instituto de Ciencias Forenses, Grupo de Medicina Xenómica (GMX), Facultade de Medicina, Universidade de Santiago de Compostela, Galicia, Spain; 2 Grupo de Investigación en Genética, Vacunas, Infecciones y Pediatría (GENVIP), Hospital Clínico Universitario and Universidade de Santiago de Compostela (USC), Galicia, Spain; 3 Fundación Pública Galega de Medicina Xenómica-SERGAS, Grupo de Medicina Xenómica-USC, IDIS, Santiago de Compostela, Galicia, Spain; 4 Infectious Diseases and Vaccines Unit, Department of Pediatrics, Hospital Clínico Universitario de Santiago, Santiago de Compostela, Galicia, Spain; University of Perugia, Italy

## Abstract

**Background:**

The origin of the Etruscan civilization (Etruria, Central Italy) is a long-standing subject of debate among scholars from different disciplines. The bulk of the information has been reconstructed from ancient texts and archaeological findings and, in the last few years, through the analysis of uniparental genetic markers.

**Methods:**

By meta-analyzing genome-wide data from The 1000 Genomes Project and the literature, we were able to compare the genomic patterns (>540,000 SNPs) of present day Tuscans (*N* = 98) with other population groups from the main hypothetical source populations, namely, Europe and the Middle East.

**Results:**

Admixture analysis indicates the presence of 25–34% of Middle Eastern component in modern Tuscans. Different analyses have been carried out using identity-by-state (IBS) values and genetic distances point to Eastern Anatolia/Southern Caucasus as the most likely geographic origin of the main Middle Eastern genetic component observed in the genome of modern Tuscans.

**Conclusions:**

The data indicate that the admixture event between local Tuscans and Middle Easterners could have occurred in Central Italy about 2,600–3,100 years ago (y.a.). On the whole, the results validate the theory of the ancient historian Herodotus on the origin of Etruscans.

## Background

The Etruscan civilization refers to the ancient culture inhabiting the territories of Etruria in the Early Iron Age, approximately 700 to 200 BC (see Beekes [1] for an explanation on the origin of the Etruscan name). The Etruria was a region of Central Italy located in an area that covered most of what now are Tuscany, Latium, and Umbria. Most of the Etruscan legacy today consists of tombs, grave goods and other material remains. Ancient historians described the Etruscan people and theorized about their origins. One of the most popular theories comes from Herodotus [2]; he proposed that the Etruscans emigrated from Lydia in Asia Minor (modern Turkey) around 1,200 BC as the result of a famine. In words of Beekes [1] “*Herodotus says that the Etruscans came from Lydia. The question is whether this is correct. My answer is: yes, but the Lydians lived at that time (also) in another area. The question of the origin of the Etruscans is one of the most debated problems of antiquity. Nowadays most scholars are convinced that they came from Asia Minor (Turkey); only in Italy does a large number of scholars deny or doubt this. The eastern origin seems certain to me, for reasons that I will present below (2.i). However, an important part of the problem has not been solved: where exactly in Asia Minor did they come from, and was this in Lydian territory, as Herodotus says?*”. Other historians, such as the Hellenistic scholar Dionysius of Halicarnassus (30 BC) contended that the Etruscans were an indigenous population that developed *in situ* from the so-called Villanovan culture (autochthonous theory). In the same line, the archaeologists Barker & Rasmussen [3] supported a local development with Eastern cultural influences in the Etruscans. The origin of the Etruscans has been the subject of intense debate among historians, and many of them are very skeptical about Herodotus's theories [4].

Genetics has recently contributed to our understanding of the origin of Etruscans through the analysis of DNA markers from present-day people from Tuscany and ancient DNA recovered from Etruscans or their presumable descendants. Most of these studies have targeted the mitochondrial DNA (mtDNA) molecule, in particular portions of its control region. One of the pioneering genetic studies, Francalacci et al. [5], pointed to an intermediate position of Tuscan mtDNAs between sequences from culturally or geographically isolated regions of Europe and those from the Middle East. More recently, Vernesi et al. [6] analyzed the mtDNA control region from 27 bone samples from Etruscans; their results indicated that the similarity between the Etruscan and Turkish gene pools may indeed reflect some degree of gene flow. However, a follow-up study by Belle et al. [7] based on simulations indicated a weak genealogical relationship between the 27 Etruscans and the modern Tuscans previously reported by Francalacci et al. [5]. By analyzing the mtDNA of 322 subjects sampled in three areas of Tuscany, Achilli et al. [8] found a high frequency of Middle Eastern mtDNA haplogroups in the locality of Murlo (a small town of Etruscan origin) while other locations in Tuscany also showed a high prevalence of haplotypes shared with Middle Easterners. By way of analyzing several *Bos Taurus* breeds from Tuscany, Pellecchia et al. [9] found that “*both humans and cattle reached Etruria from the Eastern Mediterranean area by sea. Hence the Eastern origin of Etruscans, first claimed by the classic historians Herodotus and Thucydides, receives strong independent support*”. The study by Guimaraes et al. [10] analyzed the mtDNA of 27 medieval Tuscans, and the results indicated genealogical discontinuities among Etruscan, medieval and contemporary Tuscans. Brisighelli et al. [11] identified, by sequencing entire mitogenomes, a novel mtDNA lineage, which was termed U7a2a (now re-named as U7b1 in Phylotree Build 16; www.phylotree.org) which could be dated to 2,300 y.a., and noted that approximately 10% of the Tuscans in their sample actually belong to this branch of the typical Middle Eastern haplogroup U7 – thus adding further support to the results by Achilli et al. The recent study by Ghirotto et al. [12] on ancient DNA samples indicated that the genetic links between Tuscany and Anatolia date back to at least 5,000 y.a., suggesting that the Etruscan culture developed locally and not as an immediate consequence of immigration from the Eastern Mediterranean shores.

When all the genetic evidence obtained so far are taken together, it seems clear that the Etruscans cannot be regarded as ancestral of all modern-day Tuscans; however, almost all the studies agree that there is a proportion of their mtDNA pool that could be traced to somewhere in the Middle East, thus testifying to an ancient connection between both regions. While the age of the Middle Eastern founding U7b1 lineage [11] would fit well with Herodotus's theory, estimates based on mtDNA analysis in Ghirotto et al. [12] (and most recently also in Tassi et al. [13]) would indicate that this genetic link is too old and therefore consistent with the development of the Etruscan culture locally – and not directly mediated by migration from the Middle East.

The present study aims to further investigate the controversial biological origin of the Etruscans by analyzing genome-wide SNP data (>540.000 SNPs) from 98 Tuscans genome-wide genotyped within The 1000 Genomes Project (http://www.1000genomes.org; [14]).

## Results

**Figure 1:**
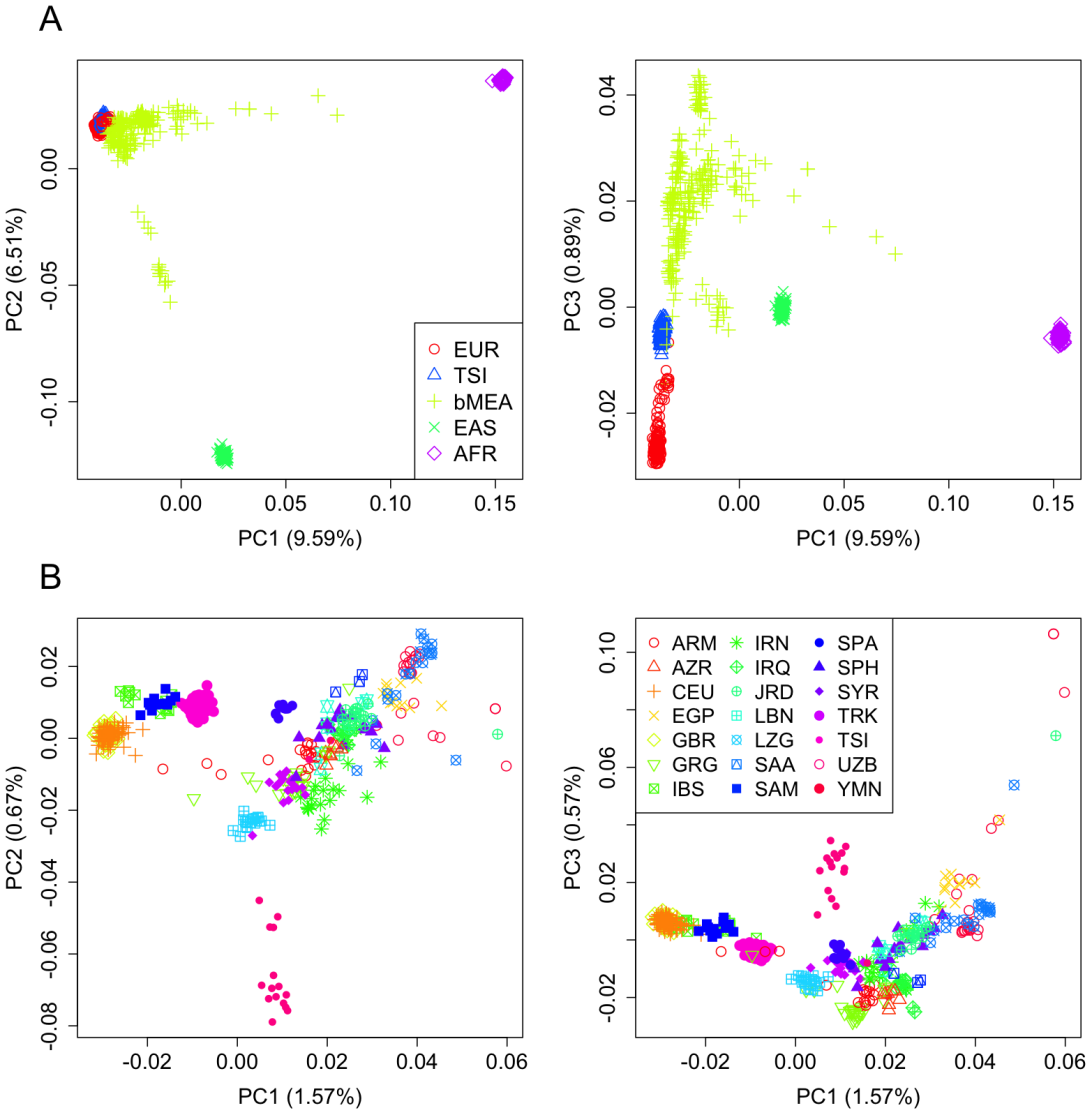
PCA of TSI and samples representing main continental groups, including Africa (AFR; represented by YRI), East Asia (EAS; represented by CHB), broad Middle East (bMEA), and Europe (EUR; represented by CEU+GBR+IBS+SPA) (Figure 1A) and PCA comparing only TSI, EUR and different populations from bMEA (Figure 1B). For each PCA analysis, only three principal components are represented (PC1 *vs.* PC2, and PC1 *vs.* PC3). See Table 1 for more information on population codes.

IBS values were computed for all the genome population datasets (**Table 1**). Firstly, PCA was carried out on the IBS values computed on Tuscans (TSI) and the main continental regions, including datasets mainly representing Europe (EUR), Sub-Saharan Africa (AFR), East Asia (EAS), and broad Middle East (bMEA); **Figure 1**. The PCA (**Figure 1A**) indicates that Tuscans are located in the European pole, perfectly separated from the African (PC1) and the East Asian nodes, a pattern that is particularly prominent in the PC2. PC3 is very informative in allowing a clear differentiation between EUR and ‘broad’ MEA (bMEA), with TSI in between; it also shows substantial scatter in bMEA compared to the other groups, probably reflecting a higher diversity in bMEA than in TSI and EUR. The PCA also shows the Tuscans to be closely related to, but clearly differentiated from, the European reference group (EUR). A second PCA was performed in order to explore the relationships of the Tuscans and the other European population samples used in the present study (**Figure S1A**); PC1 separates Central Europeans (GBR+CEU) from Tuscans in two well-differentiated clusters and occupying two opposite poles of the plot; in between fall the Iberians (IBS and SPA) and North Italians (NIT). This analysis indicates the distinctive nature of the Tuscans in the context of Europe, even when compared to other neighboring North Italians. Corroborating evidence comes from further exploring patterns of IBS values between European population sets, **Figure S1B** shows that IBS values are statistically different when comparing TSI against North Italians (Wilcoxon test; *P*-value = 1.31×10^−7^); while other comparisons such as CEU *vs*. GBR are not statistically significant (*P*-value = 0.981) (**Figure S1C**); which is consistent with the PCA analysis (**Figure S1A**). A third PCA was carried out in order to further explore the genomic affinities of Tuscans with the bMEA and Europe (**Figure 1B**). The PCA shows Tuscans in between Middle Easterners and Europeans; PC1 reveals TSI in close proximity to the populations in the Caucasus, CAU, including Armenians (ARM) and Georgians (GRG), and Lezgins (LZG), followed by a mix of several other groups in the MEA, including Turks (TRK), Sephardi Jews (SPH; sampled in Turkey), and West Asia (Uzbeks; UZB), although the PC2 clearly differentiates LZG and UZB from TSI and the other groups. The relationship between TSI and other populations in the bMEA is unclear given the limited structure of these populations along the PC1. The PC2 of **Figure 1B** shows SPH, Samaritans (SAM) and other groups as more closely related to TSI than TRK. The PCA displayed in **Figure S2** helps to understand these population relationships by highlighting the gravity center of the IBS values (average IBS values) for each population set.

**Table 1:** Population data used in the present study.

Population/Ethnicity	Continental region/ancestry	Acronym	*N*	Source
CEPH individuals	USA (Utah residents of European ancestry)	CEU	85	[14]
British	England and Scotland; Europe	GBR	56	[14]
Finnish	Finland; Europe	FIN	93	[14]
Spanish individuals	Spain; Europe	IBS	14	[14]
Spanish individuals	Spain; Europe	SPA	12	[18]
Tuscan individuals	Italy (Tuscany); Europe	TSI	98	[14]
North Italians (non-Tuscan)	Italy (Bergamo); Europe	NIT	13	[15]
Yoruba individuals	Nigeria (Ibadan); Africa	YRI	88	[14]
Han Chinese	China (Beijing); East Asia	CHB	97	[14]
Armenians	Armenia; Caucasus	ARM	19	[18]
Georgians+Georgian Jews	Georgia; Caucasus	GRG	20+4	[18]
Iranians+Iranian Jews	Iran; Middle East	IRN	20+4	[18]
Jordanians	Jordan; Middle East	JRD	20	[18]
Lebanese	Lebanon; Middle East	LBN	7	[18]
Syrians	Syria; Middle East	SYR	16	[18]
Turks	Turkey; Middle East	TRK	19	[18]
Azerbaijani Jews	Azerbaijan; Caucasus	AZR	8	[18]
Egyptians	Egypt; North Africa	EGP	12	[18]
Iraqi Jews	Iraq; Middle East	IRQ	11	[18]
Lezgins	Russia, Caucasus	LZG	18	[18]
Saudis	Saudi Arabia; Arabia	SAA	20	[18]
Samaritians	Israel; Middle East	SAM	3	[18]
Sephardi Jews	Turkey; Middle East	SPH	10	[18]
Uzbeks+Uzbekistani Jews	Uzbekistan; West Asia	UZB	15+2	[18]
Yemenis+Yemenite Jews	Yemen; Arabia	YMN	10+15	[18]

The Jews individuals included in various datasets are indicated, and the set of analysis carried out without using Jews genetic profiles are presented in **Text S1**.

**Figure 2:**
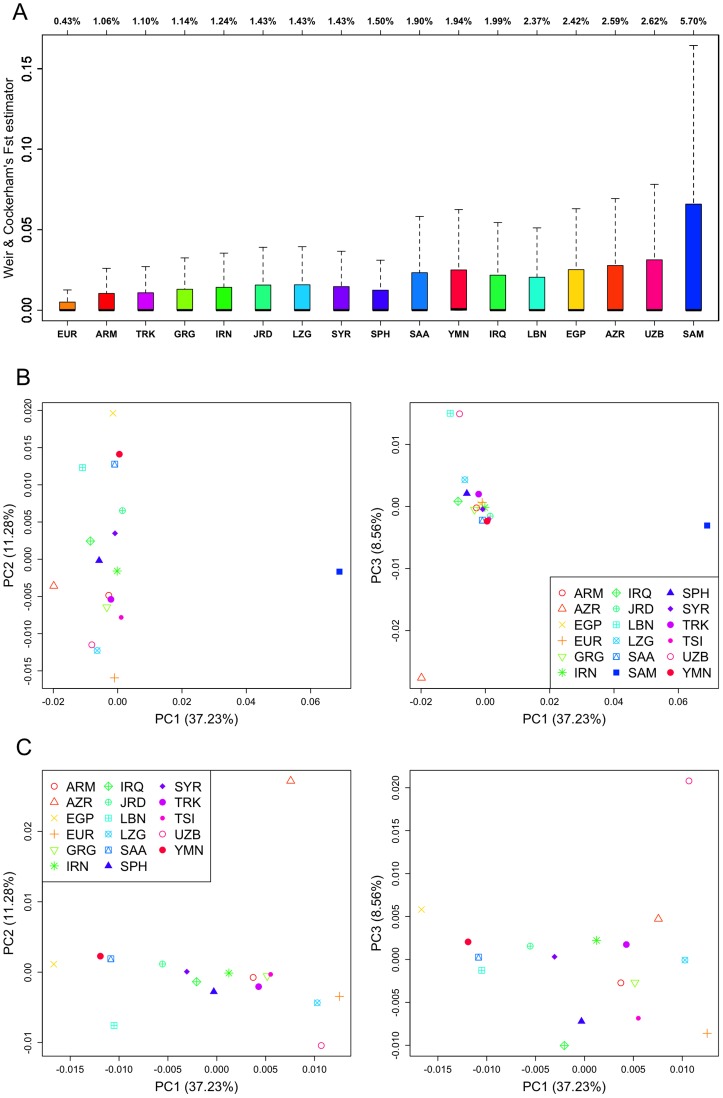
Weir and Cockerham's individual SNP F_ST_ values (the whiskers range from the lowest datum still within 1.5 IQR of the lower quartile [equals to 0 in this case], and the highest datum still within 1.5 IQR of Q3) between TSI *vs*. EUR and populations from bMEA (Figure 2A). The populations are sorted from the lowest to the highest average F_ST_ values against TSI (indicated for each population in the top of each F_ST_ distribution). PCA plots of individual SNP F_ST_ values considering all populations from bMEA (**Figure 2B**), and the same PCA after eliminating SAM from the analysis (**Figure 2C**).

F_ST_ values between TSI and all the other population groups from Europe and bMEA are shown in **Figure 2A**. The lowest F_ST_ values are for the population pair TSI-EUR, followed by the pairs TSI-ARM, TSI-GRG, TSI-IRN, TSI-SPH, TSI-LZG, and TSI-SYR, and TSI-TRK (**Figure 2A**). As also observed when examining IBS values, ARM, GRG, LZG and SPH (followed by TRK) appear as more closely related to TSI than other populations in bMEA. A PCA plot of F_ST_ values (**Figures 2B and 2C**) indicates a tight genetic relationship of TSI with GRG, ARM, and TRK, in both the PC1 and PC2; this plot separates these four groups from the rest of bMEA; EUR, however, falls close to this cluster on both the PC1 and, more clearly, on the PC2. **Figure S3** shows the frequency distributions of F_ST_ values for TSI against EUR and all populations in bMEA. The histograms help to explain the patterns observed in the PCA carried out on F_ST_ values; thus, ARM, GRG and TRK are the populations in bMEA showing the lowest values of F_ST_ and the differences with other populations are more evident when looking at the range of F_ST_ values between 0.05 and 0.40.

**Figure 3:**
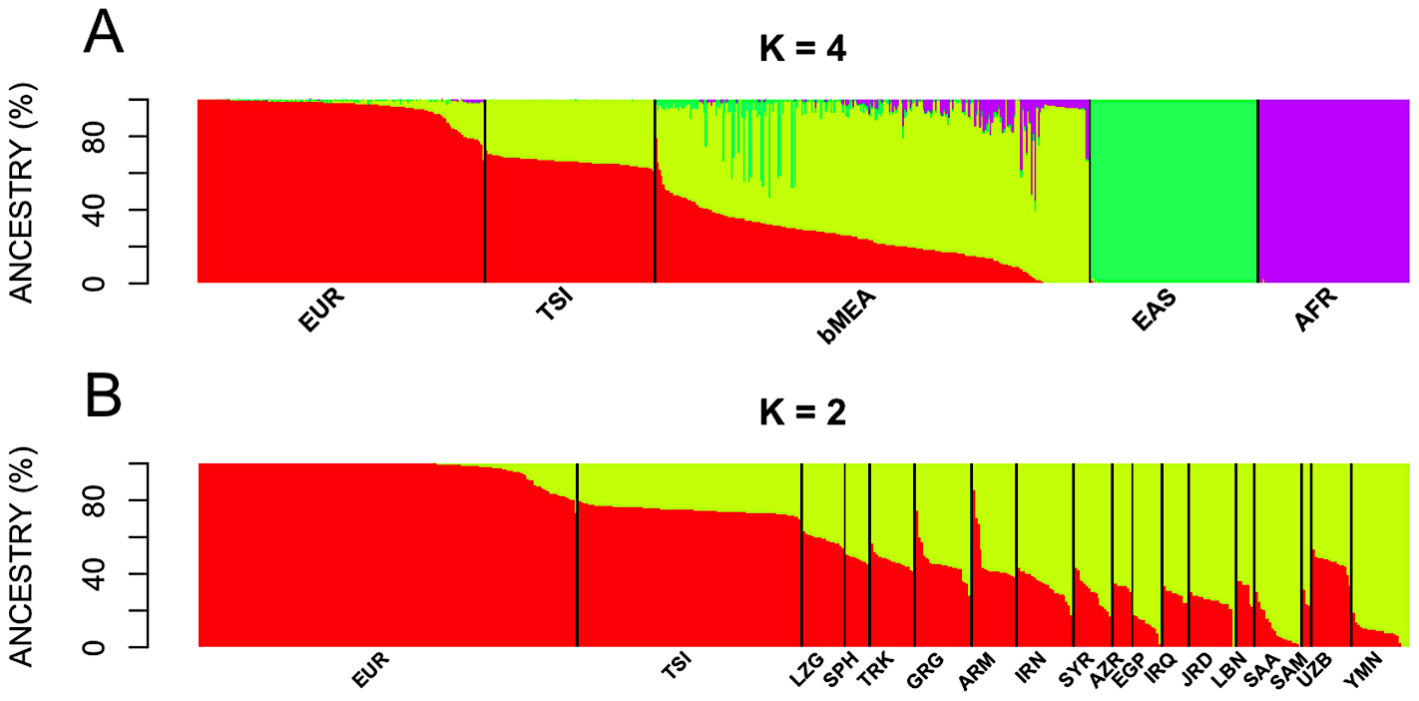
Bar-plot of individual ancestries as computed using the unsupervised clustering algorithm implemented in ADMIXTURE. Figure 3A considered TSI and main continental groups, AFR, EAS, EUR, and bMEA, with *K* = 4 being the lowest cross validation value. Figure 3B considered TSI against EUR and the different populations from bMEA, with *K* = 2 as the optimal cross validation value.

Admixture analysis was carried out using the software ADMIXTURE, and the results were represented as bar-plots in **Figure 3**. When examining TSI ancestry against main continental groups (**Figure 3A**), the results agree well with those observed in the PCA plots, indicating a clear differentiation of the main continental groups. Thus, for *K* = 4 (*K* indicating the number of inferred clusters showing the lowest error cross validation error) the results clearly reveals a major differentiation of AFR, CHB, bMEA and EUR, but also the admixture nature of TSI regarding EUR and bMEA. A second analysis of ADMIXTURE was carried out for EUR, TSI, and different bMEA populations considering *K* = 2 (**Figure 3B**); the results show again TSI as an admixed profile between EUR and bMEA, but they do not show a clear structure within bMEA populations. This analysis also reveals a shared African component of YMN, SAA and EGP. Other ADMIXTURE runs considering different *K* values are shown in **Figure S4**. On average, ADMIXTURE indicates that TSI has a large European component when compared to main continental groups (∼66%; **Table 2**) and when compared to Europe and bMEA populations (∼75%; **Table 3**), but it also has a prominent Middle Eastern component (34% and 25%, respectively [average: 30%] **Tables 2** and **3**). The populations in the bMEA with the largest European component are LZG (59%), SPH (48%), and TRK (57%); **Table 3**.

**Table 2:** Admixture proportions in ADMIXTURE analysis as carried out on main continental groups.

	bMEA	East Asia	Europe	Sub-Saharan Africa
**EUR**	4.0 (6.2)	0.5 (0.6)	95.3 (6.4)	0.2 (0.4)
**TSI**	33.7 (2.1)	0.0 (0.1)	66.3 (2.1)	0.0 (0.0)
**bMEA**	68.0 (17.8)	4.8 (9.7)	23.3 (14.6)	3.8 (7.3)
**EAS**	0.1 (0.3)	99.9 (0.4)	0.0 (0.2)	0.0 (0.0)
**AFR**	0.0 (0.2)	0.9 (0.9)	0.0 (0.0)	100 (0.2)

Values correspond to average admixture proportions in **Figure 3A**. Standard deviations are given in brackets.

**Table 3:** Admixture proportions obtained from ADMIXTURE as carried out on EUR and different populations from the MEA.

	European ancestry	Middle Eastern ancestry
EUR	97.4 (5.5)	2.6 (5.5)
TSI	74.6 (1.9)	25.4 (1.9)
LZG	58.7 (2.5)	41.3 (2.5)
SPH	48.1 (1.7)	51.9 (1.7)
TRK	47.1 (3.5)	52.9 (3.5)
GRG	45.4 (9)	54.6 (9)
ARM	46.7 (13)	53.3 (13)
IRN	33.6 (6.4)	66.4 (6.4)
SYR	29.8 (7.8)	70.2 (7.8)
AZR	33.1 (1.5)	66.9 (1.5)
EGP	12.3 (4.4)	87.7 (4.4)
IRQ	28.9 (2.7)	71.1 (2.7)
JRD	24.3 (6.2)	75.7 (6.2)
LBN	31.2 (5.9)	68.8 (5.9)
SAA	9.9 (8.6)	90.1 (8.6)
SAM	25.9 (4.6)	74.1 (4.6)
UZB	45.9 (4.4)	54.1 (4.4)
YMN	7.8 (4.5)	92.2 (4.5)

Values correspond to average admixture proportions in **Figure 3B**. Standard deviations are given in brackets.

**Figure 4:**
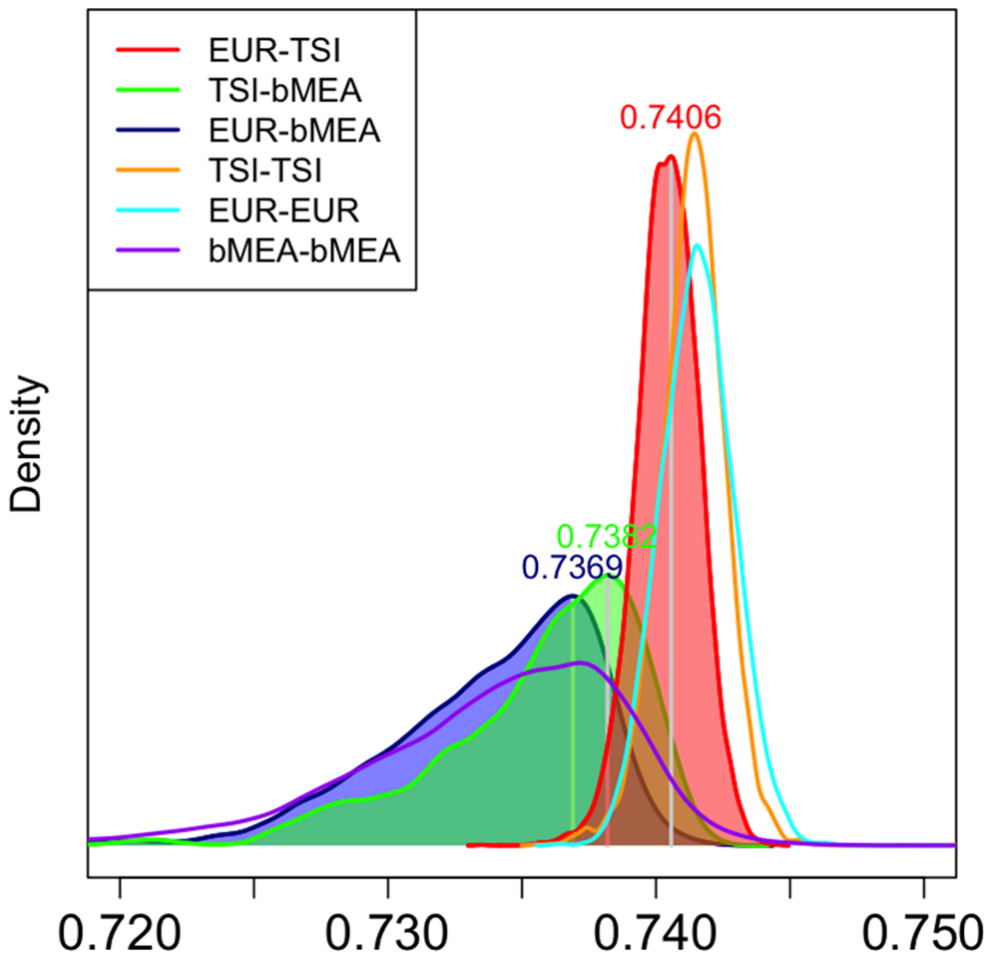
Density curves of intra- and inter-population IBS values for EUR, bMEA and TSI.

In order to study in more detail the relationship between TSI and the different populations in bMEA, we further explored patterns of IBS in these populations. **Figure 4** displays the density functions of IBS values calculated for all possible combinations of EUR, TSI and different bMEA populations. When looking at the intra-population patterns of IBS values, EUR-EUR and TSI-TSI show similarly high IBS values when compared to bMEA-bMEA, reflecting closer genomic relationships among European individuals than among individuals within the Middle East. Moreover, bMEA shows a wider and more heterogeneous range of IBS values. Of the three possible inter-population comparisons, the pair EUR-bMEA shows the lowest IBS values (reflecting the largest genetic distance out of the three possible inter-population comparisons), while TSI shows a pattern that is in between intra-population IBS variation of bMEA-bMEA and EUR-EUR.

**Figure 5:**
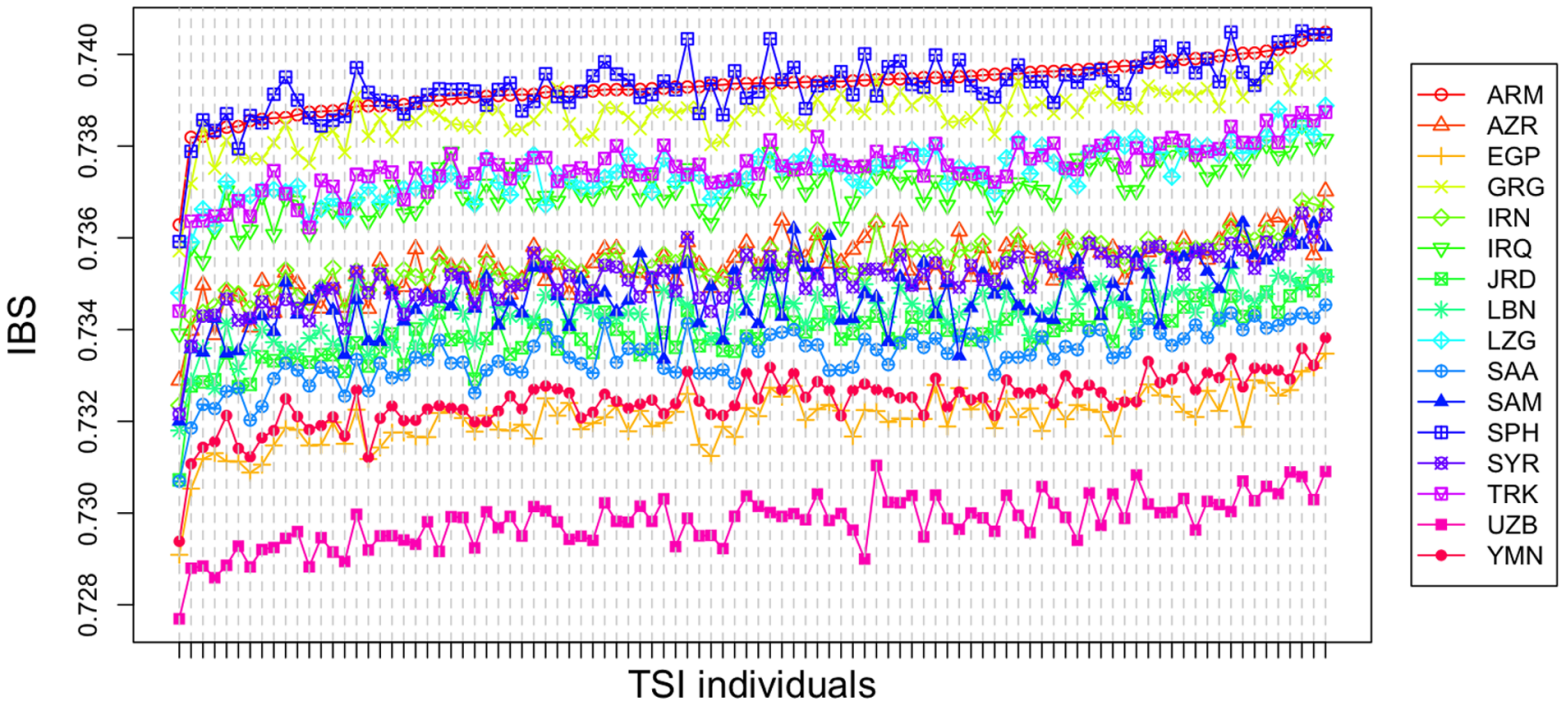
Average IBS values between TSI individuals and individuals from EUR and other bMEA populations. IBS values were arranged from the lowest averages with TSI (namely, ARM) to the highest.

IBS values were also computed for each TSI individual against each population in bMEA (**Figure 5**). This analysis very clearly indicates the highest values for TSI individuals when compared against ARM, followed by SPH, GRG and TRK. The populations showing the lowest IBS values with TSI are UZB, YMN, and EGP.

**Figure 6:**
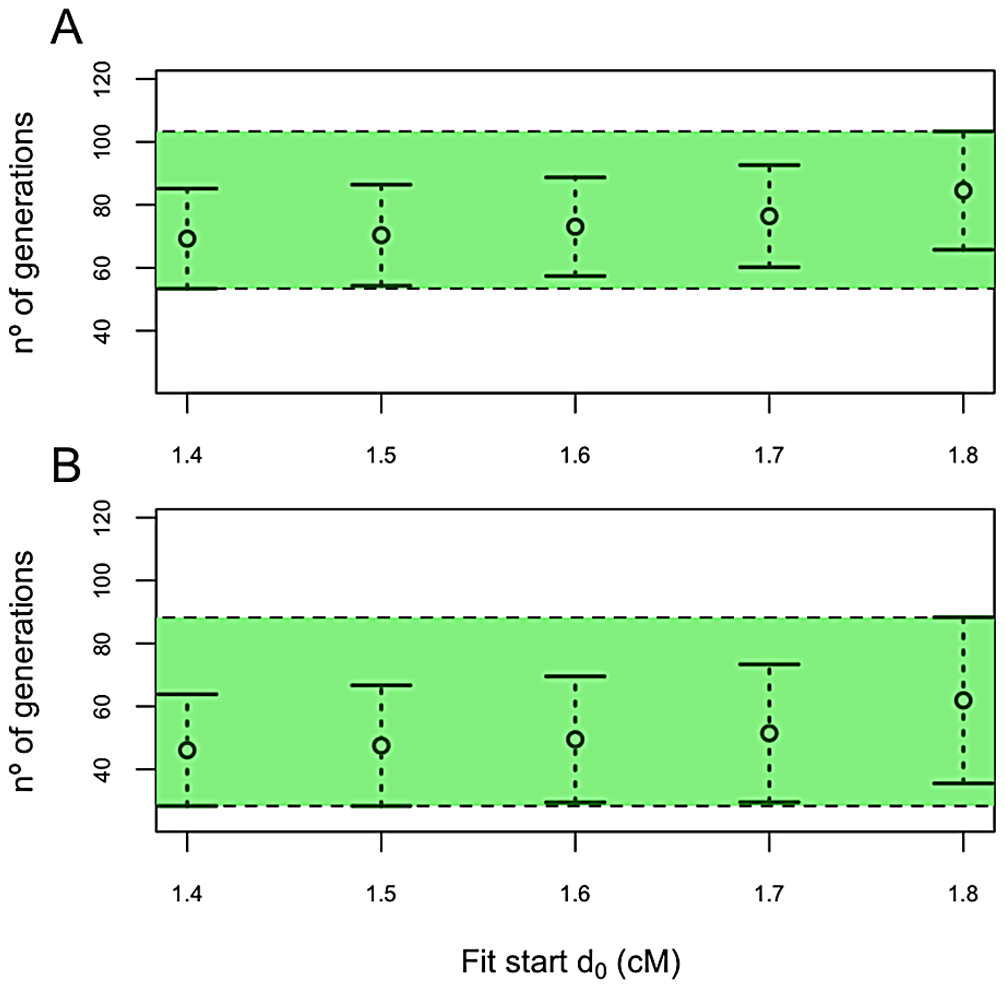
Estimates of the age of admixture in TSI considering EUR and bMEA as the two source populations (7A) and considering EUR and a sample that merges the datasets from Turkey (TRK, SHP) and South Caucasus (ARM, GRG, AZR, and LZG). All the estimates were statistically significant under the *ad hoc* z test carried out by ALDER.

We used ALDER to estimate the date of admixture between TSI and bMEA populations. The first analysis was carried out using bMEA. ALDER yields a range of age for the admixture event with bMEA of 53–103 generations (average: 78), which considering a generation age of 30 years, equates to 1,590–3,090 y.a. (average: 2,340 y.a.); **Figure 6**. To circumvent the uncertainties about the exact ancestral source, we conducted the same analysis using the different samples from bMEA as surrogate ancestral populations; however, the results were inconsistent or statistically insignificant probably due to their small sample sizes. We then proceeded to merge in a single sample the most likely source populations contributing to the Middle Eastern component of TSI as suggested by the previous analysis (that is, Turkey and the Caucasus). ALDER yields a range of age for the admixture event of 28–88 generations (average: 58), which equates to 840–2,640 y.a. (average: 1,740 y.a.); **Figure 6**. Taken into account the two age estimates obtained from ALDER, and more importantly, the fact that age estimates represent an underestimate of the real admixture event, it seems reasonable to consider the upper range of both admixture events as a reasonable range for the admixture event, that is, between 2,600 and 3,100 y.a. When excluding the Jews profiles from the analysis the ranges are slightly larger, and the upper bound reaches 3.535 y.a. (**Text S1**)

ALDER also estimates the membership proportions in TSI with respect to the source populations. The contribution from Middle East to TSI when comparing to bMEA is 26.2% (±7.9), but it drops to 21.1% (±8.4) when using the most likely source populations from bMEA (Turkey and South Caucasus). When excluding the Jews profiles from the analysis (**Text S1**), these values range from 26.7 (±9.0; comparing to bMEA) to 18 (±8.4; against Turkey and South Caucasus); 22% on average. These estimates are comparable to the values obtained using ADMIXTURE (∼30%).

## Discussion

The present study aimed to add further insight into the origin of the Etruscans by analyzing present-day Tuscans, which are assumed to be at least in part their descendants. There are several lines of evidence in the literature indicating that the DNA of modern populations in Tuscany as well as DNA from ancient Etruscans have genetic signatures of a recent historical ancestry from the Middle East. All of this evidence, however, comes from the analysis of uniparental markers (mtDNA). The present study goes beyond previous work in that it presents the first genome-wide based approach aimed to disentangle the origin of Tuscans and their presumable genetic connections with the ancient Etruria and the Middle East. The few Tuscans analyzed before by Chaubey et al. [15] were intended to be part of other analysis and the resolution of other historical/biological dilemmas. However, it was also evident in their admixture analysis that their Tuscans carried a distinctive Middle Eastern character. Interestingly, some (non-Tuscan) North Italians were also incorporated in their analysis, and their Figure 3 barplot of ancestries suggested some similarity with Tuscans, a feature that is expected if we assume bidirectional gene flow between Tuscany and its most neighboring region. The analyses performed in the present study (**Figure S1**) indicate that these few North Italians differentiate from Tuscans but the analysis is also compatible with recent gene flow between them.

Admixture analyses carried out on autosomal SNPs corroborates the admixture nature of Tuscans between Europe and the Middle East; with values of Middle Eastern ancestry of about 22–30%; this component is substantially larger than that previously detected using mtDNA data (5–10% on average [8], [11], with a peak of 18% in the Tuscan village of Murlo [8]). Admixture bar-plots do not allow enough resolution to determine which population in the Middle East is most closely related to TSI. However, PCA of IBS values indicates that TSI occupies an intermediate genetic position between Europe and the Middle East, and that it also shows affinities to various population groups in the bMEA, including ARM, GRG, SPH and TRK. When examining patterns of IBS values between TSI and bMEA populations in more detail (**Figure 5**), the results point to ARM, followed by SPH (sampled in Turkey), GRG and TRK, as the best source populations in bMEA explaining this variation in TSI. Supporting evidence comes from the analysis of genetic distances (F_ST_ values), which point to a closer genetic proximity of TSI with the CAU (GRG, ARM), and Turkey (TRK and SPH). The fact that the main non-European signal in Tuscan genomes comes from CAU (and not from e.g. West Turkey) cannot be explained only by a geographic gradient across Eurasia related to geographic distance.

The moment where the admixture event between Europeans and Middle Easterners took place in the ancestral Etruria can be estimated from the analysis of the exponential decay of admixture-induced linkage disequilibrium as a function of genetic distance. These estimates indicate that admixture between Middle Easterners and Europeans could have occurred about 2,600–3,100 y.a.

The present study adds further support to the findings reported in previous studies based on mtDNA. On the whole, the results corroborate the presence on an important genomic bMEA component in Tuscans. Based on the fact that the population sets ARM, GRG, SPH (from Turkey) and TRK recurrently appear in most of our analyses as the most closely genetically related to TSI, it can be tentatively suggested that the most likely source population in the bMEA contributing to (at least part of) the Middle Eastern component observed in modern Tuscans (TSI) could have inhabited the territories located between Turkey and the CAU. Given the proximity of Lydia (West Turkey) to this region and the age admixture estimates obtained in the present study, the hypothesis formulated by Herodotus on the geographic origin of Etruscans could not be fully disregarded.

The present findings on admixture proportions in Tuscans fit quite well with the information extracted from the atlas of human admixture recently published by Hellenthal et al. [16]; their globetrotter tool (http://admixturemap.paintmychromosomes.com) indicates that the proportion of admixture coming from Armenia, Georgia, Syria and Jordania is 27.6% in total; being Armenia the main source population (10.7%). Their estimate for the admixture event is younger (about 1,000 years ago) than the age obtained in our study, but these estimates were obtained using other datasets (including e.g. Morocco).

## Conclusions

The dating calculated in the present study is in good agreement with the age of the Middle Eastern haplogroup U7 lineages (2,300 y.a.) identified in the Isle of Elba (Tuscany) by Brisighelli et al. [11]. However, our findings conflict with the most recent proposal of Tassi et al. [13] and Ghirotto et al. [12] based on theoretical simulations of mtDNA patterns observed in Etruscan and Medieval samples from Tuscany. Both studies suggested that the genetic links between Tuscany and Anatolia do exist, but date back to a remote stage of prehistory (at least 5,000 y.a.). On the other hand, the mtDNA data in general (both modern and ancient DNA) are compatible with a proportion of lineages coming from the Middle East, not necessarily from Turkey or South Caucasus, but also further southeast (e.g. Iran).

**Figure 7:**
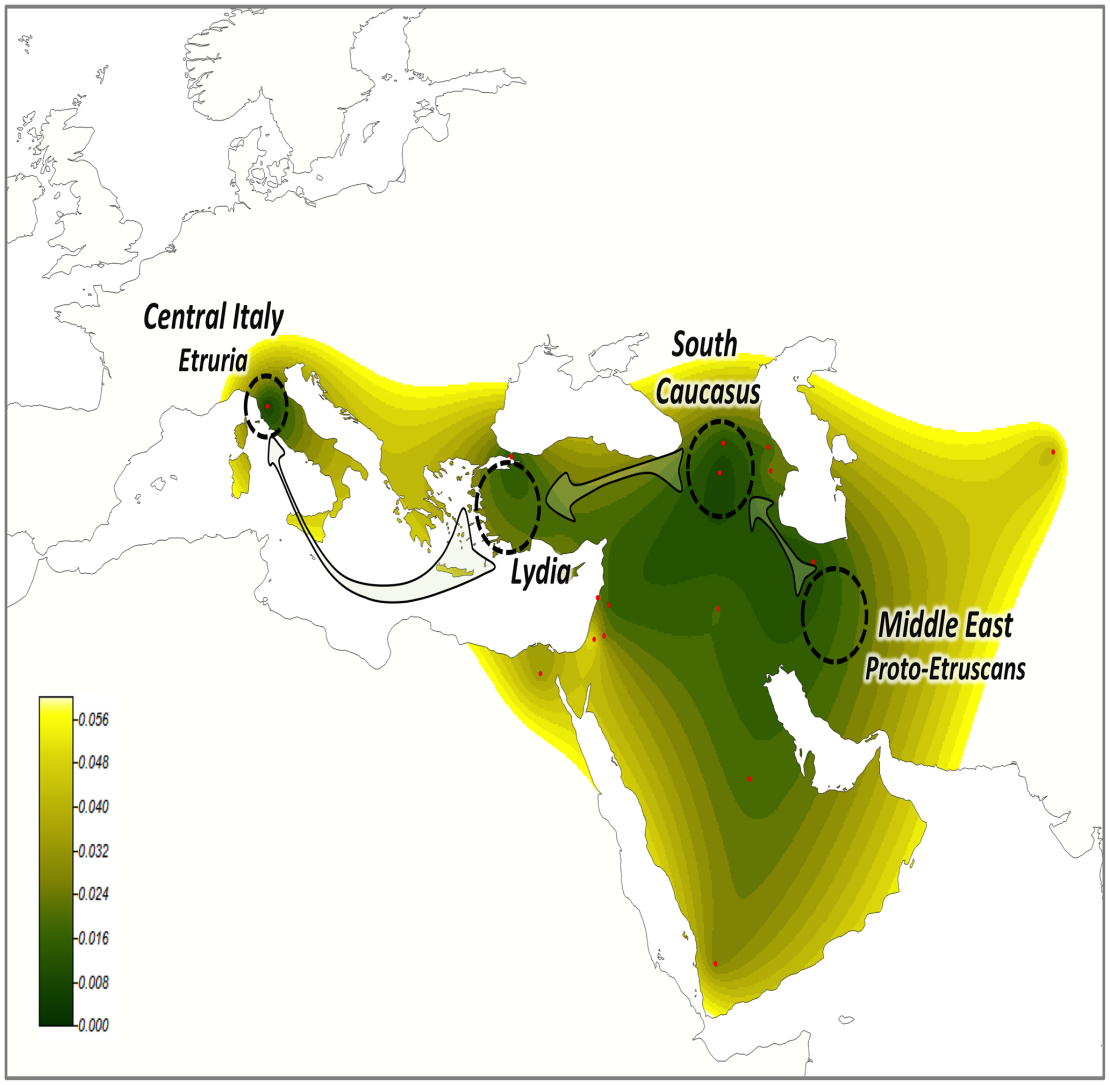
The migration model aims to explain the origin of Etruscans accommodating the different findings reported on mtDNA studies and those observed in the present study. The map shows F_ST_ interpolated values between TSI and Middle Eastern populations (no European datasets were considered here) with colors ranging from dark green (lowest values) to yellow (highest values). The non-colored map regions were not interpolated.

In order to accommodate the different findings, we propose the following multi-step demographic scenario (**Figure 7** and **Figure S5**): (i) the emergence of a distinctive population (proto-Etruscans) in south/southeast Middle East ∼5000 y.a., (ii) this small group of Middle Easterners could have travelled westwards to a region located in South Caucasus/East Turkey and from here further West crossing Anatolia and arriving at Lydia (West Turkey); this process would involve an interval of time allowing for assimilation of genetic features from indigenous people, and (iii) this population could have left the West Mediterranean shores and journeyed towards Central Italy before 2,600–3,100 y.a. The Etruscan culture could have emerged at about the time of arrival of these Middle Easterners. This model integrates the different elements observed in the genomes of present-day Tuscans: notable in features of the mtDNA variation inherited from the Proto-Etruscan sources, and a clear signature from South Caucasus and Turkey in their patterns of autosomal variation.

Considering the recorded as well as the likely unrecorded population flows within Europe in the last couple of thousand years, the study of the Etruscans using modern DNA of their most presumable descendants, the Tuscans, is very challenging. For instance, the date obtained in the present study for the admixture estimates could actually represent a mixture of two European populations ∼2,600–3,100 y.a., and not necessarily a mixture of a European and a Middle Eastern population. If these hypothetical populations had different proportions of Middle Eastern ancestry, their mixture would generate a strong linkage disequilibrium curve when using present-day Europeans and present-day Middle Easterners as proposed sources. The Middle Eastern genomic patterns observed in present-day Tuscans could also be the result of various overlapping waves of migrants coming from different regions in Middle East and South Caucasus at different times; some of them could have arrived to this region in Neolithic times.

Genome-wide SNP analysis of ancient DNA obtained from Etruscans (in the same line as those previously done in other demographic contexts, e.g. [17]), would be most helpful for the exploration of these potential alternative demographic scenarios and the deconvolution of the different historical demographic movements that may have contributed to the genome of modern Tuscans. Furthermore, future genetic studies on modern DNA should also focus on a more detailed sampling of Middle Eastern populations, Turkey and southern Caucasus in particular, ideally using higher density SNP panels, thus allowing for higher genome resolution.

## Methods

### Data-mining from The 1000 Genomes Project data

Genome population data were downloaded from two different repositories. SNP data representing different Middle Eastern populations were firstly obtained from Behar et al. [18]. The rest of the population data were taken from The 1000 Genomes Project. For the bioinformatic treatment of The 1000 Genomes Project data (http://ftp.1000genomes.ebi.ac.uk/vol1/ftp/phase1/data), we took advantage of previous bioinformatic developments [19], [20]. Thus, all variant files were firstly downloaded from the 1000Genomes project public ftp (ftp://ftp.1000genomes.ebi.ac.uk/vol1/ftp/release/20110521/). All the rs codes defined on Behar et al.'s article [18] were saved in LGEN format, which could be loaded into PLINK [21] to get the binary BED file that would be used for subsequent analysis. The MAP file needed was generated by cutting the first four columns in the BIM SNP file of Behar et al.'s study, and the FAM file needed was created by listing all The 1000 Genomes Project samples and adding missing code “-9” on all columns as required. Although the downloaded genome data contained more than 37.5 million SNPs [20], the overlapping data from all the population datasets used in this study consisted of 542,305 SNPs. The present study focuses on the Middle Eastern component of Tuscans, and this is the reason why we have incorporated a large set of potential source populations from this region. The CEU dataset along was initially used in the present project to capture the European component from Tuscans, given that Central Europeans, better than e.g. Iberians, could appear as a more appropriate surrogate ancestral population for present-day Tuscans [16]. We observed however that the incorporation of different European samples into the statistical models yielded virtually the same results in regards to the relationship of Tuscans with the Middle East. Therefore, in order to better represent the variability from Europe, we finally decided to use different available population datasets from Europe, including, CEU, IBS, GBR (collected from The 1000 genome Project) and the Spaniards (SPA) from Behar et al. [18]; altogether merged in a single sample are referred in the text as EUR. The YRI and CHB datasets were used as representative of sub-Saharan Africa (AFR) and East Asia (EAS), respectively. The few (*n* = 13) North Italians analyzed in Chaubey et al. [15] were also used to prove the distinctive genetic character of Tuscans in North Italy and with respect to other European populations (**Figure S1**); this analysis involved 542,305 SNPs (as a result of merging NIT with the other Europeans).

Individuals having a familial relationship were detected as done in Gómez-Carballa et al. [22] and eliminated from the analysis (see **Text S1**). A summary of the populations used in the present study is given in **Table 1**. All the statistical analyses were also computed disregarding the Jewish from the Behar's et al. study; the results were fully consistent with the ones shown in the main text and are shown in **Text S1**.

PLINK was also used to deal with strand differences between SNP sets. For some analyses and for the sake of simplicity, the Middle Eastern population sets (including South Caucasus) were grouped in a single group, namely, “broad Middle East” (bMEA).

### Statistical analysis of autosomal data

Identity-by-state (IBS) values were computed from SNP data using PLINK [21]. Weir and Cockerham's F_ST_ estimator [23] was computed from SNP data and on all pairwise population comparisons using VCFtools [24].

In order to discriminate clusters of genetic variation in the population sets analyzed, principal component analyses (PCA) were carried out on a matrix of pairwise individual IBS and F_ST_ values. PCA was carried out using the function *cmdscale* (library *stats*) from R (http://www.r-project.org).

Admixture in Tuscans was estimated using different source populations in Europe and bMEA. ADMIXTURE software [25] uses a maximum likelihood estimation of individual ancestries from multilocus SNP data; this software was used to estimate percentages of admixture in Tuscans to a continental level considering main continental groups (sub-Saharan Africa, East Asia, Middle East and Europe) and to an intra-continental level considering only Europe and different populations from bMEA.

Dating of admixture was carried out using ALDER [26]. Input files for ALDER were prepared using EIGENSOFT [27]. Admixture was modeled using Europeans (EUR) and Middle Easterners (all populations merged in a single group or) as the two reference source populations. The model generated by ALDER assumes that mating occurs randomly since the initial generation of admixture, and that the process of admixture (which could be simple or multiple) gives more emphasis on more recent admixture events [28]. Therefore, it has to be acknowledged that ALDER could be underestimating the real age of the initial contact. ALDER also provides estimates on proportions of ancestry from the considered reference populations.

The spatial geographical representation of F_ST_ and IBS values between TSI and Middle Eastern populations was carried out using SAGA v. 2.1.1 (http://www.saga-gis.org/). We followed the commonly used ordinary Kriging method for interpolating F_ST_ and IBS values; other interpolated methods yielded virtually the same results.

In-house R and Perl (http://www.perl.org) scripts were used to display results obtained from the different software packages used.

## Supporting Information

Figure S1 **PCA analysis of the European population datasets used in the present study; including the few non-Tuscan North Italians employed by Chaubey et al. [15]**
(TIF)

Figure S2 **PCA carried out as in** **Figure 1** **but highlighting the gravity center for each population group (the average IBS values of each sample); individual profiles are displayed in grey color.**
(TIF)

Figure S3 **Histograms of Weir and Cockerham's F_ST_ values between TSI** ***vs*** **. CEU and different populations in MEA.** Population colors in histograms are as in the rest of the figures. The inset histograms show more detail for the distribution of F_ST_ values ranging from 0.05 and 0.40.
(TIF)

Figure S4 **Bar-plots of individual ancestries as computed using ADMIXTURE for different values of** ***K*** **considering TSI and main continental groups, AFR, EAS, EUR, and bMEA (Figure S2A) and considering TSI with EUR and the different populations from bMEA (Figure S2B).**
(TIF)

Figure S5 **The map shows IBS interpolated values between TSI and Middle Eastern populations with colors ranging from dark green (highest values) to yellow (lowest values).** The non-colored map regions were not interpolated.
(TIF)

Text S1 **Estimation of familial relationship between the individuals analyzed in the present project, and statistical analysis removing Jewish subject and considering a Finnish population, FIN, collected from The 1000 genome Project).**
(DOCX)
