# Supplementary figures and images for: A Genome-Wide Study of Modern-Day Tuscans: Revisiting Herodotus's Theory on the Origin of the Etruscans
Source: PLoS One. 2014 Sep 17;9(9):e105920. doi: 10.1371/journal.pone.0105920 (PMC4167696; doi:10.1371/journal.pone.0105920)

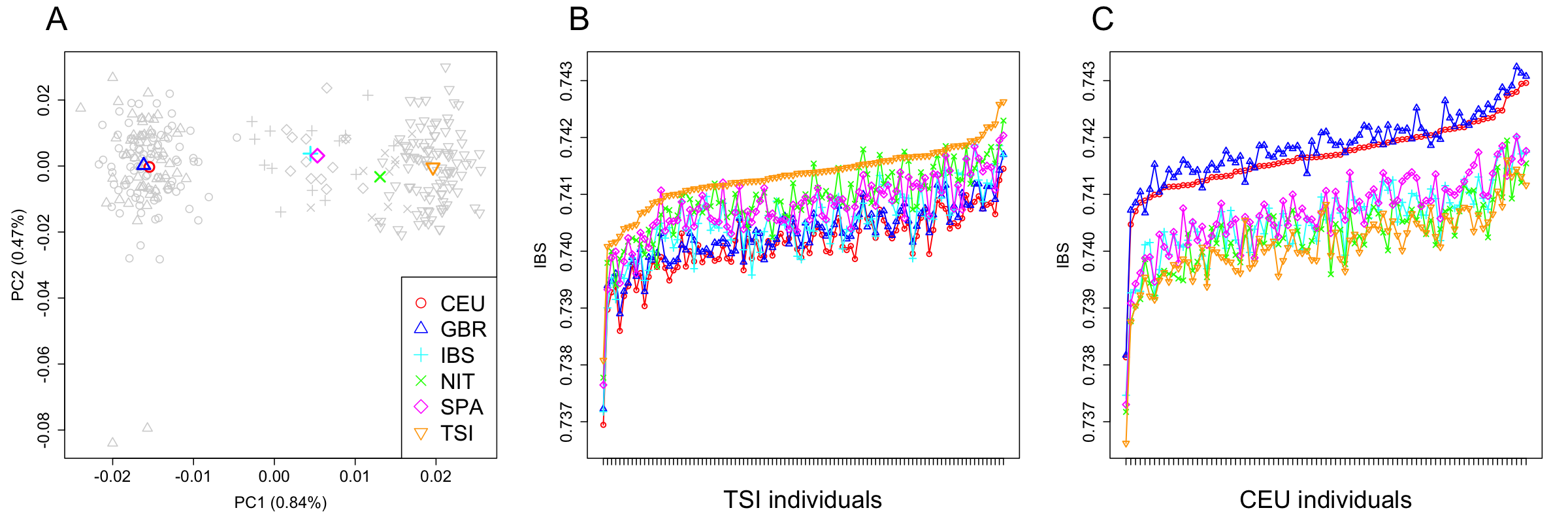

Supplement: Figure S1 — PCA analysis of the European population datasets used in the present study; including the few non-Tuscan North Italians employed by Chaubey et al. [15] (TIF) [file pone.0105920.s001.tif]

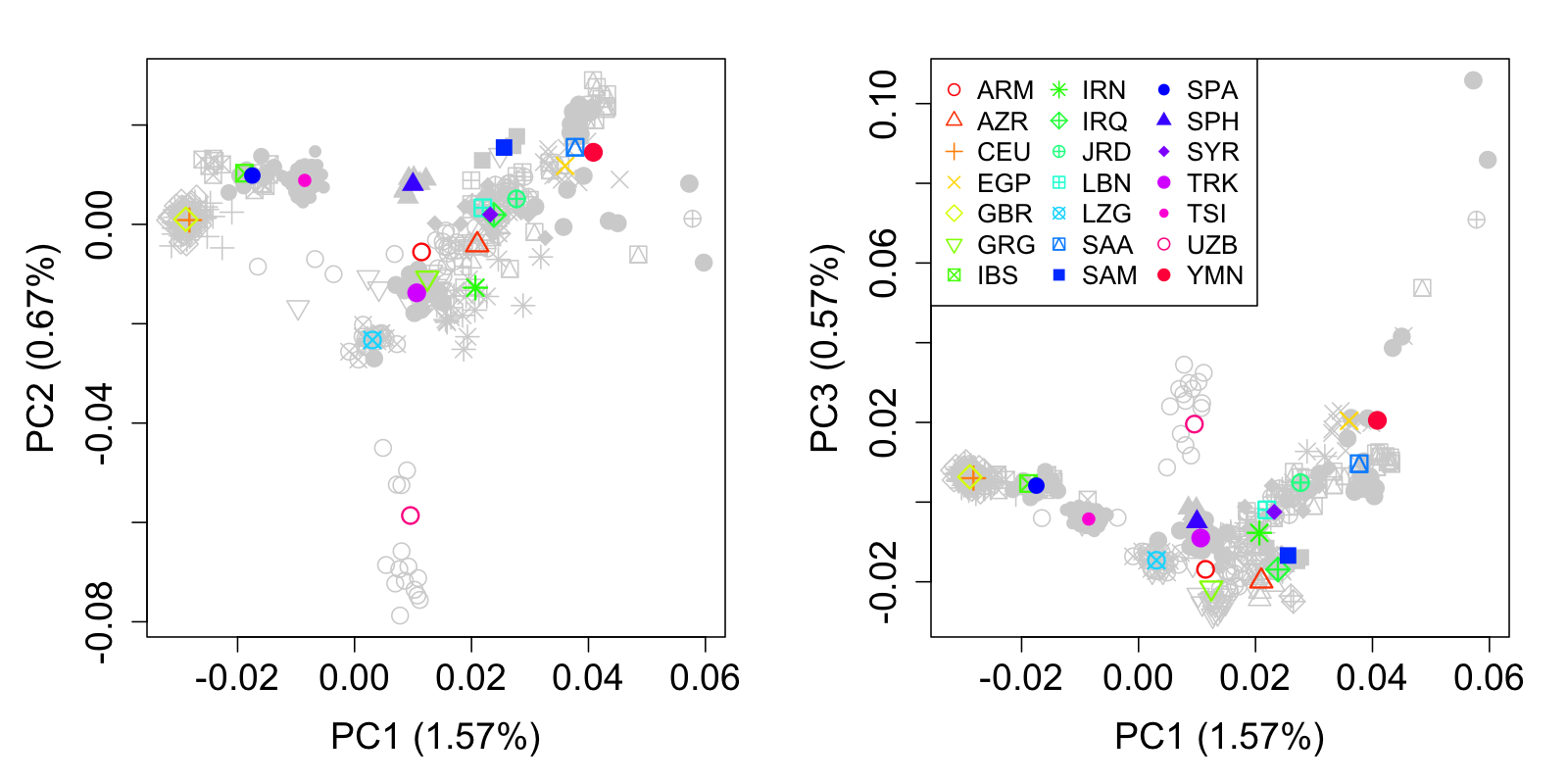

Supplement: Figure S2 — PCA carried out as in Figure 1 but highlighting the gravity center for each population group (the average IBS values of each sample); individual profiles are displayed in grey color. (TIF) [file pone.0105920.s002.tif]

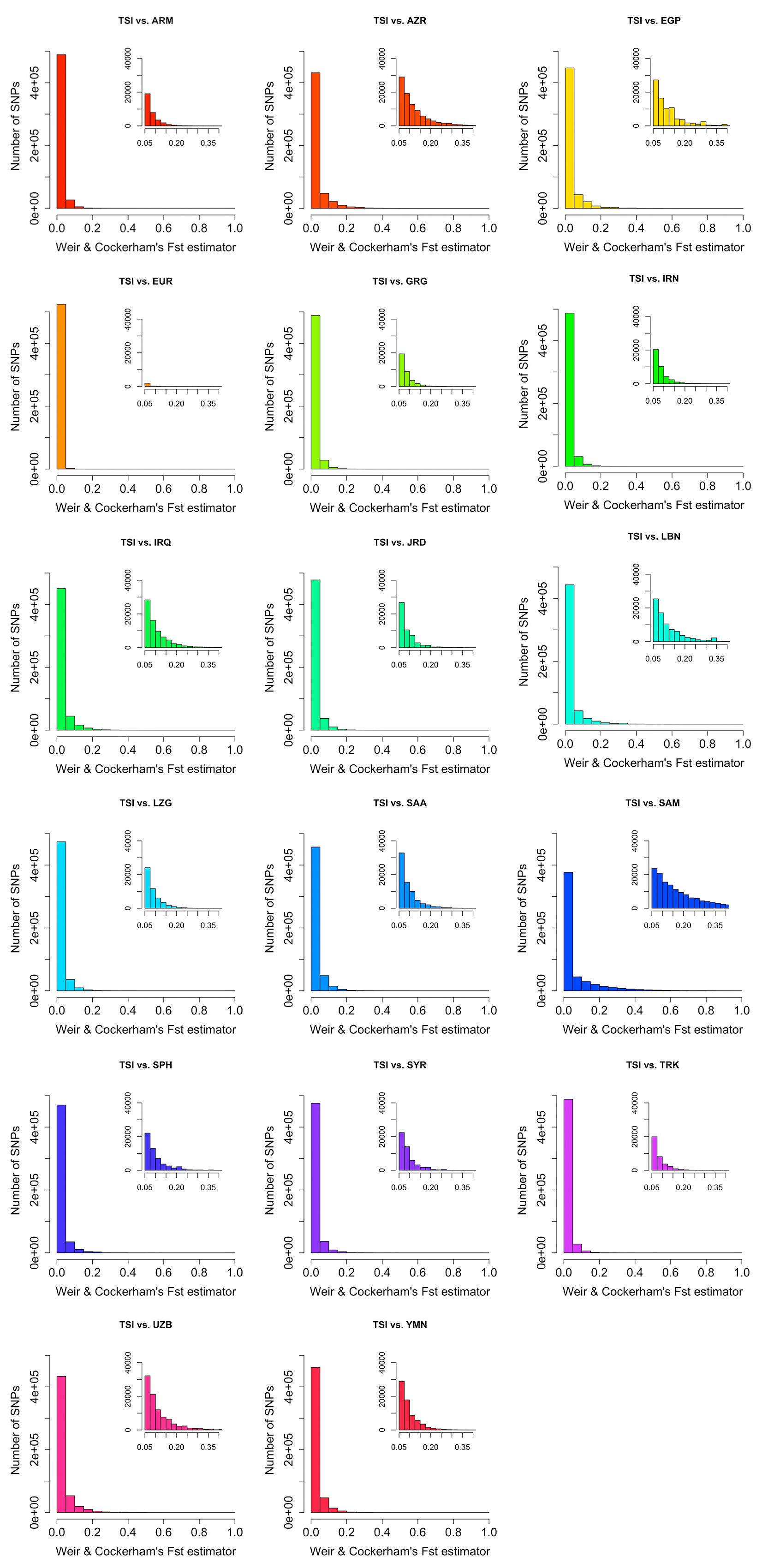

Supplement: Figure S3 — Histograms of Weir and Cockerham's FST values between TSI vs . CEU and different populations in MEA. Population colors in histograms are as in the rest of the figures. The inset histograms show more detail for the distribution of FST values ranging from 0.05 and 0.40. (TIF) [file pone.0105920.s003.tif]

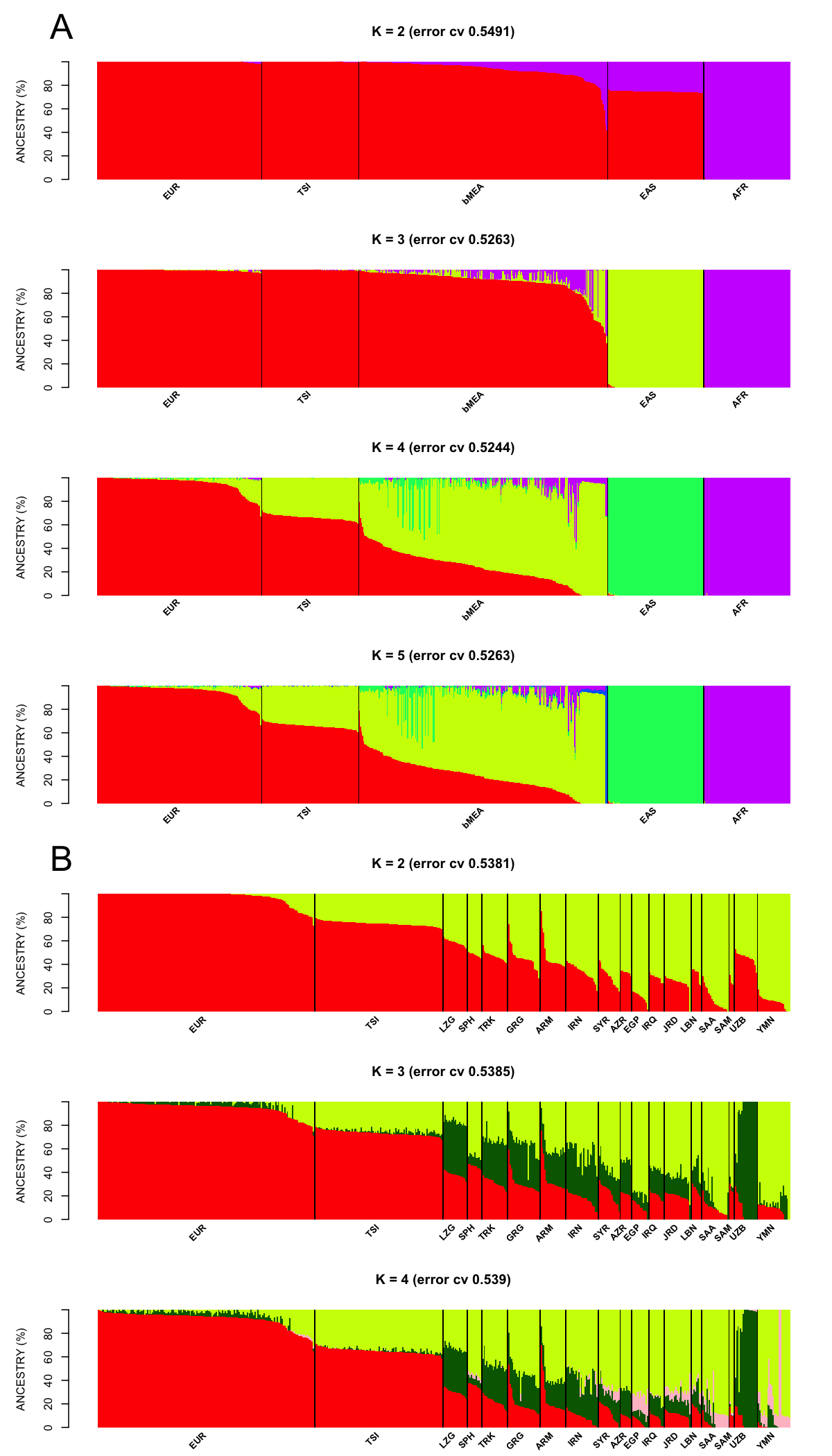

Supplement: Figure S4 — Bar-plots of individual ancestries as computed using ADMIXTURE for different values of K considering TSI and main continental groups, AFR, EAS, EUR, and bMEA (Figure S2A) and considering TSI with EUR and the different populations from bMEA (Figure S2B). (TIF) [file pone.0105920.s004.tif]

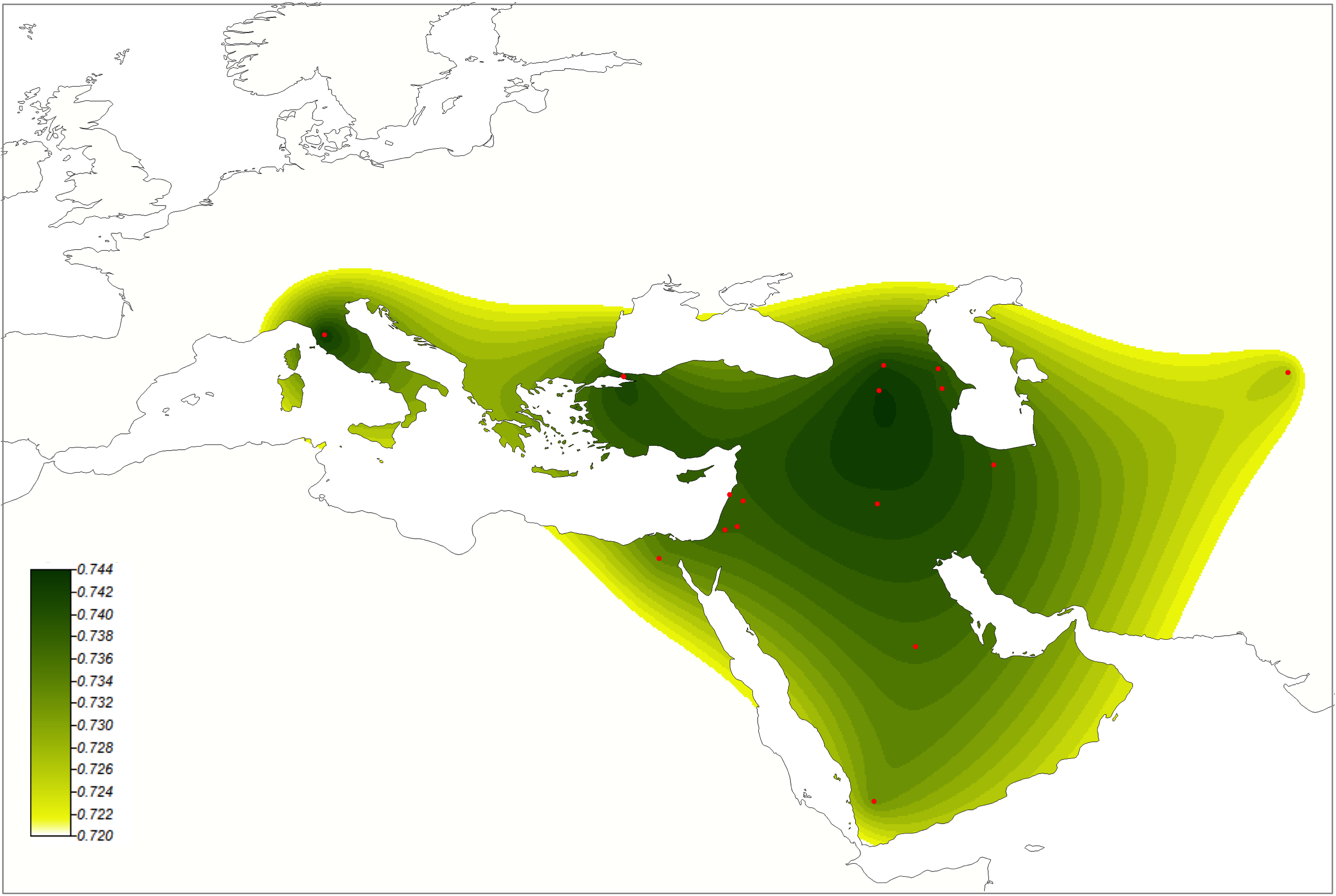

Supplement: Figure S5 — The map shows IBS interpolated values between TSI and Middle Eastern populations with colors ranging from dark green (highest values) to yellow (lowest values). The non-colored map regions were not interpolated. (TIF) [file pone.0105920.s005.tif]
