# Supplementary material for: A Genome-Wide Study of Modern-Day Tuscans: Revisiting Herodotus's Theory on the Origin of the Etruscans
Source: PLoS One. 2014 Sep 17;9(9):e105920. doi: 10.1371/journal.pone.0105920 (PMC4167696; doi:10.1371/journal.pone.0105920)
Supplement: Text S1 — Estimation of familial relationship between the individuals analyzed in the present project, and statistical analysis removing Jewish subject and considering a Finnish population, FIN, collected from The 1000 genome Project). (DOCX) [file pone.0105920.s006.docx]

**Text S1**

This **Text S1** file contains **Table S1** and a block of analysis carried out excluding Jews from all the datasets.

| **Individual 1** | **Individual 2** | ***k_0_*** | ***k_1_*** | ***k_2_*** | ***k_1_*/2+*k_2_*** | **IBS2 ratio** | **Relationship** |
| --- | --- | --- | --- | --- | --- | --- | --- |
| NA20786 (TSI) | NA20811 (TSI) | 0.869 | 0.124 | 0.007 | 0.069 | 0.705 | <3º |
| Yemen_Jew_3 (YMN) | Yemen_Jew_4 (YMN) | 0.881 | 0.091 | 0.029 | 0.074 | 0.708 | <3º |
| NA11931 (CEU) | NA11933 (CEU) | 0.844 | 0.140 | 0.016 | 0.086 | 0.707 | <3º |
| NA20516 (TSI) | NA20816 (TSI) | 0.838 | 0.128 | 0.035 | 0.098 | 0.707 | <3º |
| NA11932 (CEU) | NA12383 (CEU) | 0.776 | 0.213 | 0.011 | 0.118 | 0.725 | <3º - 3º |
| HG00238 (GBR) | HG00142 (GBR) | 0.712 | 0.270 | 0.018 | 0.153 | 0.754 | 3º |
| HG00134 (GBR) | HG00240 (GBR) | 0.702 | 0.278 | 0.021 | 0.160 | 0.763 | 3º |
| Samaritian988 (SAM) | Samaritian990 (SAM) | 0.478 | 0.424 | 0.097 | 0.309 | 0.820 | 2º |
| iran10 (IRN) | iran20 (IRN) | 0.283 | 0.522 | 0.196 | 0.457 | 0.905 | Full sibling |
| Yemen7 (YMN) | Yemen2 (YMN) | 0.286 | 0.481 | 0.233 | 0.473 | 0.897 | Full sibling |
| iraq_jew3 (IRQ) | iraq_jew7 (IRQ) | 0.002 | 0.946 | 0.053 | 0.525 | 0.998 | Parent-child |
| Yemen6 (YMN) | Yemen3 (YMN) | 0.000 | 0.000 | 1.000 | 1.000 | 1.000 | Identical twins |

**Table S1**. Estimation of familial relationship between the individuals analyzed in the present project. Relationships were estimated using the parameters *k_0_*, *k_1_* y *k_2_* that represent the proportion of IBD shared alleles. See also [[1](#_ENREF_1)]. The results fit with those in Table 1 of Stevens et al. [[2](#_ENREF_2)]. The relationships shown in Table S1 are statistically significant using the test proposed by Lee [[3](#_ENREF_3)] and applying a multiple test correction.

**Figure 1 from main text without Jews**. Fins were also incorporated in this Figure but disregarded from all the analysis (see main text).

**
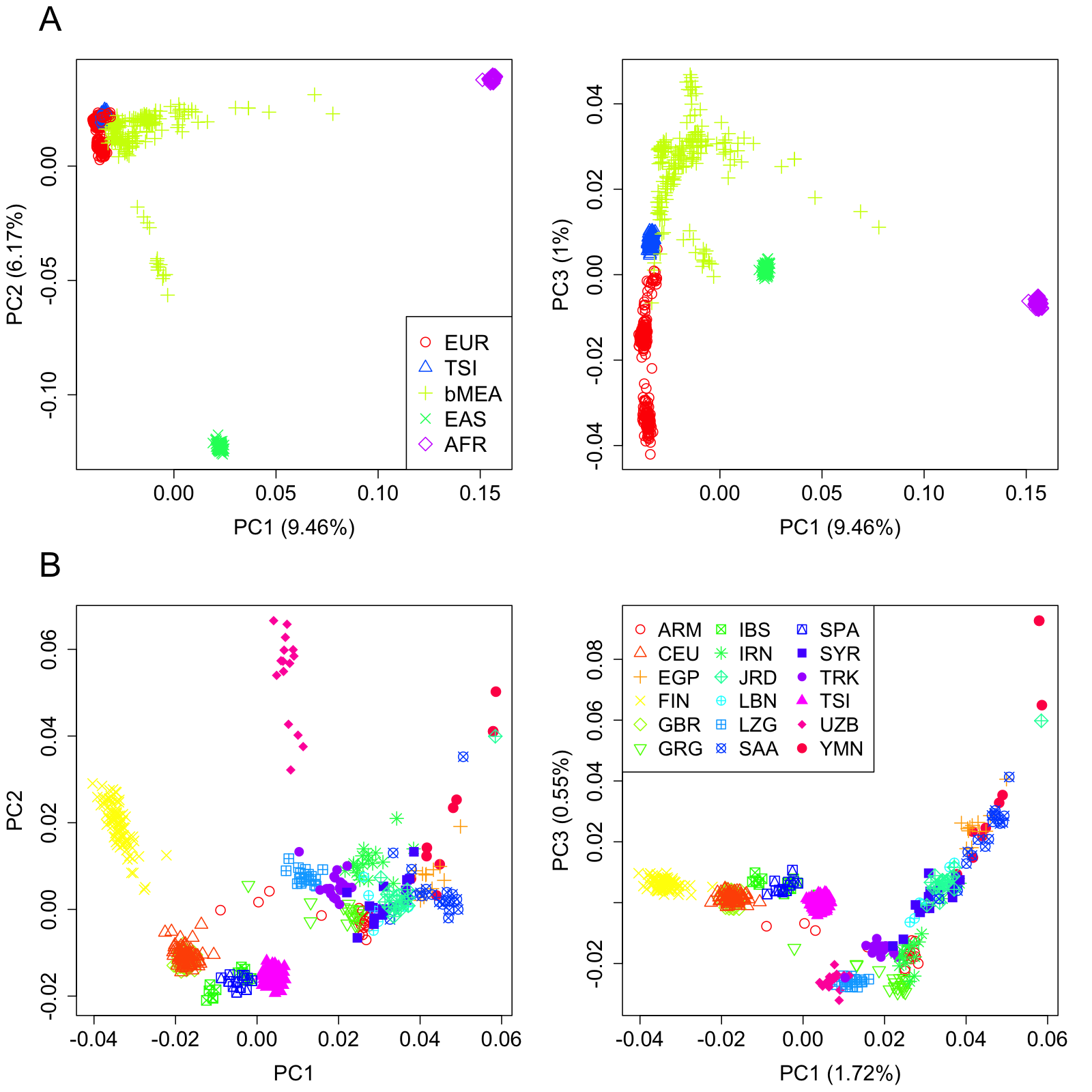
**

**Figure 2 from main text without Jews.**

**
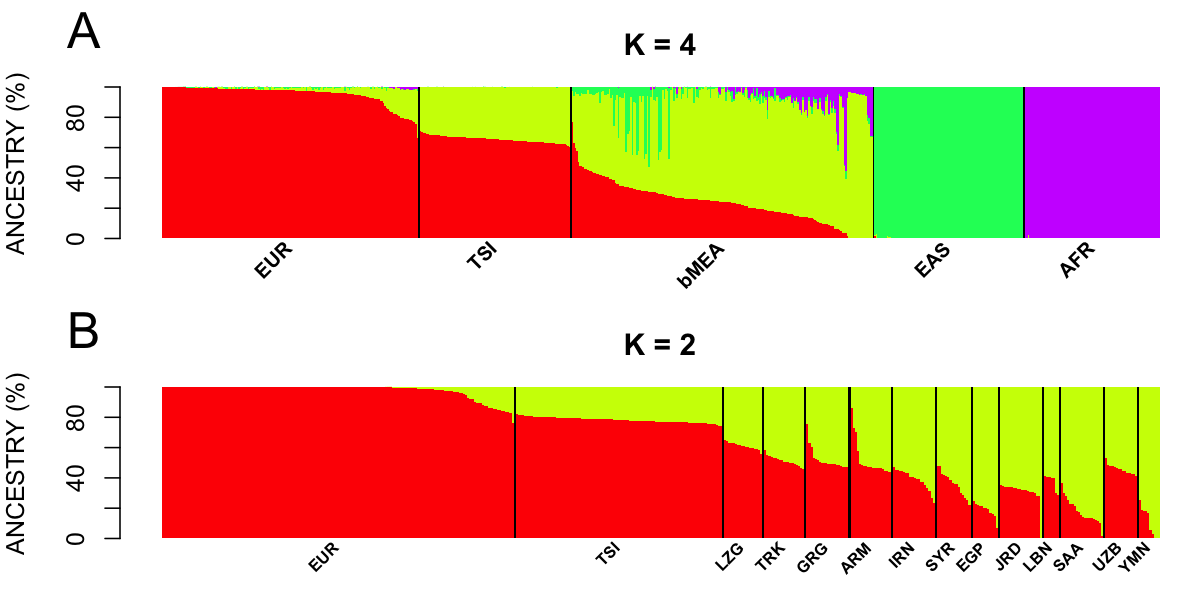
**

**Figure 6 from main text without Jews**. The upper and lower 95%CI bounds using bMEA are 46.89-117.87 (A), whereas using CAU are 24.96-101.52.

**
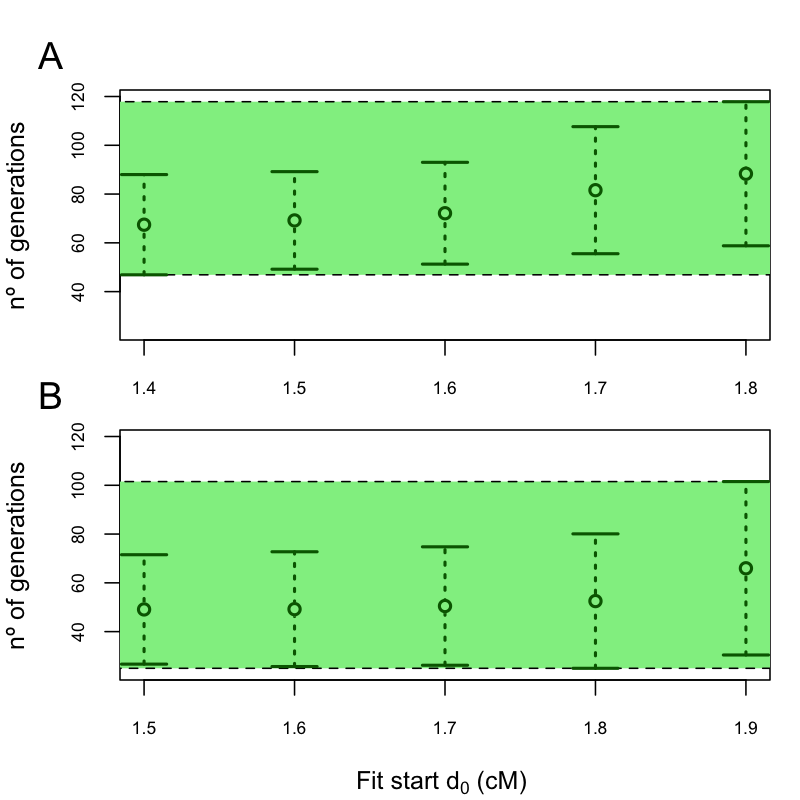
**

**Table 2 from main text without Jews.**

|  | **Middle Eastern** | **East Asian** | **European** | **Sub-Saharan Africa** |
| --- | --- | --- | --- | --- |
| **EUR** | 4.2 (6.1) | 0.5 (0.6) | 95.2 (6.4) | 0.2 (0.5) |
| **TSI** | 34.5 (2.1) | 0 (0.1) | 65.5 (2.1) | 0.0 (0.0) |
| **bMEA** | 65.9 (16.9) | 5.9 (10.7) | 23.5 (13.6) | 4.6 (8.1) |
| **EAS** | 0.1 (0.3) | 99.9 (0.4) | 0.0 (0.2) | 0.0 (0.0) |
| **AFR** | 0.0 (0.2) | 0.0 (0.0) | 0.0 (0.0) | 100 (0.2) |

**Table 3 from main text without Jews.**

|  | **European ancestry** | **Middle Eastern ancestry** |
| --- | --- | --- |
| **EUR** | 97.8 (4.7) | 2.2 (4.7) |
| **TSI** | 78.2 (1.7) | 21.8 (1.7) |
| **LZG** | 61.1 (2.3) | 38.9 (2.3) |
| **TRK** | 51.1 (3.2) | 48.9 (3.2) |
| **GRG** | 52.0 (6.8) | 48.0 (6.8) |
| **ARM** | 51.9 (11.5) | 48.1 (11.5) |
| **IRN** | 39.1 (6.4) | 60.9 (6.4) |
| **SYR** | 36.0 (7.8) | 64.0 (7.8) |
| **EGP** | 18.9 (4.7) | 81.1 (4.7) |
| **JRD** | 30.6 (7.5) | 69.4 (7.5) |
| **LBN** | 37.4 (5.4) | 62.6 (5.4) |
| **SAA** | 17.8 (8.1) | 82.2 (8.1) |
| **UZB** | 45.7 (3.0) | 54.3 (3.0) |
| **YMN** | 10.5 (9.8) | 89.5 (9.8) |

**References**

1. Hedrick PW (2011) Genetics of populations. Canada: Jones and Bartlett Publishers.

2. Stevens EL, Heckenberg G, Roberson ED, Baugher JD, Downey TJ, et al. (2011) Inference of relationships in population data using identity-by-descent and identity-by-state. PLoS Genet 7: e1002287.

3. Lee WC (2003) Testing the genetic relation between two individuals using a panel of frequency-unknown single nucleotide polymorphisms. Ann Hum Genet 67: 618-619.
